# Stimulatory effects of novel glucosylated lactose derivatives GL34 on growth of selected gut bacteria

**DOI:** 10.1007/s00253-018-9473-8

**Published:** 2018-11-07

**Authors:** Hien T. T. Pham, Markus C. L. Boger, Lubbert Dijkhuizen, Sander S. van Leeuwen

**Affiliations:** 1 Microbial Physiology, Groningen Biomolecular Sciences and Biotechnology Institute (GBB), University of Groningen, Nijenborgh 7, 9747 AG Groningen, The Netherlands; 2 Present Address: CarbExplore Research B.V, Zernikepark 12, 9747 AN Groningen, The Netherlands; 3 Present Address: Department of Laboratory Medicine, University Medical Center Groningen, University of Groningen, 9713 GZ Groningen, The Netherlands

**Keywords:** Glucosylated lactose derivatives (GL34), Synbiotics, *Bifidobacterium*, *Lactobacillus*, Probiotic bacteria

## Abstract

Previously we structurally characterized five glucosylated lactose derivatives (F1–F5) with a degree of polymerization (DP) of 3–4 (GL34), products of *Lactobacillus reuteri* glucansucrases, with lactose and sucrose as substrates. Here, we show that these GL34 compounds are largely resistant to the hydrolytic activities of common carbohydrate-degrading enzymes. Also, the ability of single strains of gut bacteria, bifidobacteria, lactobacilli, and commensal bacteria, to ferment the GL34 compounds was studied. Bifidobacteria clearly grew better on the GL34 mixture than lactobacilli and commensal bacteria. Lactobacilli and the commensal bacteria *Escherichia coli* Nissle and *Bacteroides thetaiotaomicron* only degraded the F2 compound α-D-Glc*p*-(1 → 2)-[β-D-Gal*p*-(1 → 4)-]D-Glc*p*, constituting around 30% *w*/*w* of GL34. Bifidobacteria digested more than one compound from the GL34 mixture, varying with the specific strain tested. *Bifidobacterium adolescentis* was most effective, completely degrading four of the five GL34 compounds, leaving only one minor constituent. GL34 thus represents a novel oligosaccharide mixture with (potential) synbiotic properties towards *B. adolescentis*, synthesized from cheap and abundantly available lactose and sucrose.

**Electronic supplementary material:**

The online version of this article (10.1007/s00253-018-9473-8) contains supplementary material, which is available to authorized users.

## Introduction

The human gut microflora has drawn increasing attention in recent years. It constitutes a very interesting ecosystem that varies in density and functionality in the different gut compartments (Ley et al. 2006). These complex ecosystems have a significant impact on host well-being (Flint et al. 2007). Strongest interest is focused on understanding what factors cause variations in microbiota composition and how these gut bacteria modulate host health (Louis et al. 2007). Our work aims to stimulate the growth of health-promoting probiotic gut bacteria by using newly synthesized non-digestible carbohydrates, i.e., prebiotic compounds.

According to the latest definition, a prebiotic is “a substrate that is selectively utilized by host microorganisms conferring a health benefit” (Gibson et al. 2017). Recently, part of the definition was disputed, since selective stimulation of health-promoting species seems not exclusively necessary to confer health benefits (Bindels et al. 2015; Yan et al. 2018). Generally, prebiotics are carbohydrates that are not fully digested by the host. They are fermented by various commensal and health-beneficial gut bacteria, thus promoting their growth and activity which may confer health benefits upon the host (Roberfroid et al. 2010; Callaway et al. 2008). To date, the most well-known prebiotics, supported by good quality data, are human milk oligosaccharides (*h*MOS) (Bode 2012), β-galacto-oligosaccharides (GOS), β-fructo-oligosaccharides (FOS), inulin, and lactulose (Slavin 2013; Macfarlane et al. 2008). All of these prebiotics are also hydrolyzed by brush border enzymes, but not completely (Ferreira-Lazarte et al. 2017). Isomalto-/malto-polysaccharides (IMMP) (Bai et al. 2016; Rycroft et al. 2001), xylo-oligosaccharides (XOS) (Marquina et al. 2002), resistant starch (Lehmann and Robin 2007), and soy oligosaccharides also are (emerging) prebiotic oligosaccharides (Jaskari et al. 1998), although more data about their effects on gut health are still needed. Each of these prebiotic compounds may exert specific and selective effects on gut bacteria. The search for new and effective prebiotics combined with specific probiotics (synbiotics) is increasing rapidly (Pandey et al. 2015; Rastall and Maitin 2002).

Lactose-derived oligosaccharides attract much attention in view of their prebiotic potential. One example is GOS, which are synthesized from lactose by enzymatic trans-galactosylation using β-galactosidases, achieving a degree of polymerization between 3 and 10 (Van Leeuwen et al. 2016). This prebiotic has been widely studied and shown to stimulate probiotic bacteria to various extents (Macfarlane et al. 2008; Boehm and Moro 2008; Rijnierse et al. 2011). Another commercially available prebiotic in this group is lactosucrose, which is hardly utilized by human digestive enzymes and has stimulatory effects on both lactobacilli and bifidobacteria (Ohkusa et al. 1995; García-Cayuela et al. 2014). Also, the selective bifidogenic effect of 4′-galactosyl-kojibiose, corresponding to compound F2 in our GL34 mixture (Pham et al. 2017), on *Bifidobacterium breve* 26M2 has been reported (García-Cayuela et al. 2014). These results indicate that there are clear perspectives to further develop and expand this group of lactose-derived prebiotic oligosaccharides.

**Scheme 1:**
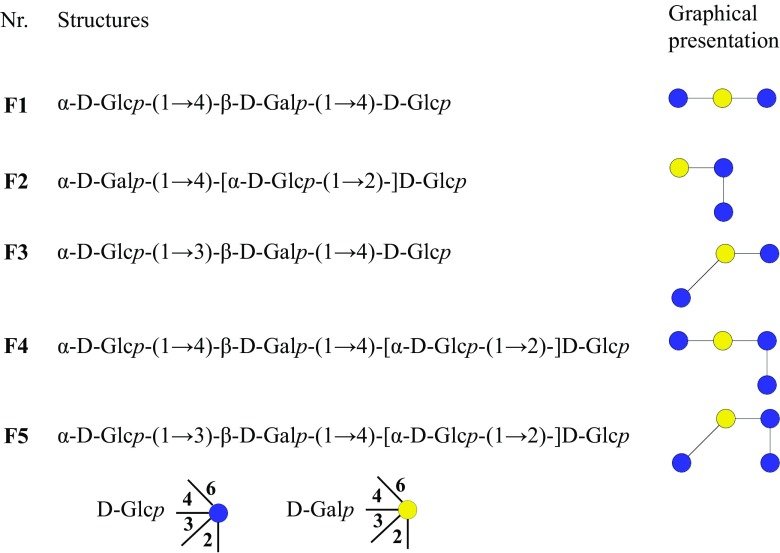
Structures of compounds F1–F5 from the mixture GL34

We recently reported synthesis of a mixture of five novel lactose-derived oligosaccharides (F1–F5) using the *Lactobacillus reuteri* glucansucrase enzymes Gtf180-ΔN and GtfA-ΔN (Pham et al. 2017). Their structural characterization revealed the presence of various glycosidic linkages, α(1 → 2/3/4), with DP of 3 and 4 (Pham et al. 2017) (Scheme 1). Four out of these five structures were new and only F2 4′-galactosyl-kojibiose had been reported before. In this work, their resistance to degradation by common carbohydrate-degrading enzymes was studied by in vitro incubations. Also, the growth of pure cultures of common gut bacteria, including commensal and probiotic strains, on these novel compounds was evaluated and compared with well-known prebiotic mixtures (GOS and FOS). The GL34 mixture particularly stimulated growth of *Bifidobacterium adolescentis*. This is also of interest from an industrial perspective, since these new oligosaccharides with very specific prebiotic effects are produced from low-cost lactose and sucrose and may be an option for developing synbiotics.

## Materials and methods

### Bacterial strains, chemicals, and reagents

*Bacteroides thetaiotaomicron* VPI-5482, *Lactobacillus acidophilus* ATCC 4356, *B. adolescentis* ATCC 15703, and *Bifidobacterium longum* subsp*. infantis* ATCC 15697 were purchased from ATCC. *B. breve* DSM 20213 was obtained from the DSMZ culture collection. *L. reuteri* strain 121 was obtained from TNO Quality of Life, Zeist, the Netherlands. *Lactobacillus casei* W56 was provided by Winclove Probiotics B.V. (Amsterdam). All reagents, chemicals, or medium components were purchased from Sigma (Zwijndrecht, Netherlands), or as stated otherwise. The purified GOS mixture TS0903 (lacking glucose, galactose, and lactose) was provided by FrieslandCampina Domo; its detailed GOS composition was published elsewhere (Lammerts van Bueren et al. 2017). The GOS/FOS mixture used is a 90:10 (*w*/*w*) mixture of the purified TS0903 GOS and long-chain Inulin (lc-Inulin, Frutafit TEX, provided by SENSUS, Roosendaal, The Netherlands), also serving as a control for the current prebiotic formula added to infant nutrition (Boehm and Moro 2008).

### *Lactobacillus* growth experiments

Lactobacilli were pre-cultured in MRS medium (Oxoid, Basingstoke, UK) anaerobically (or by using the GasPak system (Becton, Dickinson and Company, Sparks, USA)) under an N_2_ atmosphere for up to 2 days at 37 °C (Daniels and Zeikus 1975). Then, 1-mL samples of the pre-cultures were harvested by centrifugation (2500×*g*, 2 min). The bacterial pellets were washed twice with sterile 10% NaCl and diluted 25-fold in 2× modified MRS (mMRS; medium that does not contain a carbon source for Lactobacilli) (Watson et al. 2013). In separate tubes, carbohydrates were dissolved with Milli-Q water to 10 mg mL^−1^ and sterilized by filtration using 0.2-μm cellulose acetate filters (the GL34 mixture) or by autoclaving solutions (glucose). Cultures were inoculated by mixing 1:1 diluted bacterial suspensions with sterilized carbohydrate solutions in sterile microtiter plates to obtain an initial OD_600_ of 0.01. Inoculation of microtiter plates was carried out in an anaerobic glove box (Sicco, Grünsfeld, Germany) with a constant nitrogen flow, the microtiter plates sealed air tightly and transferred into a plate reader placed under constant N_2_ flow. Glucose was used as positive control to compare growth of these lactobacilli on the mixture of GL34 compounds. Media without an added carbon source was used as negative control. Bacterial growth was followed at 37 °C by measuring the optical density at 600 nm (OD_600_ nm) in 5-min intervals. OD values of the negative control samples (no carbohydrate added) were deducted while measuring their corresponding samples and positive controls.

### *Bifidobacterium* and *B. thetaiotaomicron* growth experiments

The *Bifidobacterium* strains were sub-cultured (from stocks stored at − 80 °C) in 10 mL of *Bifidobacterium* medium (BM) supplemented with 1% glucose (1-L BM contained 10 g trypticase peptone, 2.5 g yeast extract, 3 g tryptose, 3 g K_2_HPO_4_, 3 g KH_2_PO_4_, 2 g triammonium citrate, 0.3 g pyruvic acid, 1 mL Tween 80, 0.574 g MgSO_4_·7H_2_O, 0.12 g MnSO_4_·H_2_O, and 0.034 g FeSO_4_·7H_2_O). After autoclaving, BM was supplemented with 0.05% (*w*/*v*) filter-sterilized cysteine-HCl (Ryan et al. 2006). *B. thetaiotaomicron* was cultured using a carbon-limited minimally defined medium of 100 mM KH_2_PO_4_ (pH 7.2), 15 mM NaCl, 8.5 mM (NH_4_)_2_SO_4_, 4 mM L-cysteine, 1.9 M hematin, 200 M L-histidine, 100 nM MgCl_2_, 1.4 nM FeSO_4_·7 H_2_O, 50 M CaCl_2_, 1 g mL^−1^ vitamin K_3_, 5 ng mL^−1^ vitamin B_12_, and individual carbon sources (0.5%, *w*/*v*) (Koropatkin et al. 2008). Carbohydrates were prepared by dissolving in Milli-Q water to 10 mg mL^−1^ and sterilized by filtration using 0.2-μm cellulose acetate filters (the GL34 mixture, GOS, FOS) or by autoclaving solutions (lactose). Growth was carried out in fermentation tubes at 37 °C under anaerobic conditions maintained by GasPak EZ anaerobe container system (BD, New Jersey, USA). Cell suspensions from overnight cultures were prepared in 3 mL of BM supplemented with different carbon source in a final concentration of 5 mg mL^−1^. BM without an added carbon source was used as negative control. OD_600_ nm measurements of the fermentation tubes were carried out manually at 1-h intervals and data used to generate growth curves. OD values of the negative control samples (no carbohydrate added) were deducted while measuring their corresponding samples and positive controls.

### *Escherichia coli* Nissle growth experiments

*Escherichia coli* Nissle was cultured using M9 medium as previously described, at 37 °C under aerobic conditions (Sambrook and Russell 2001). OD_600_ nm measurements of the fermentation tubes were carried out manually at 2-h intervals. OD values of the negative control samples (no carbohydrate added) were deducted while measuring their corresponding samples and positive controls.

### Enzyme incubations

The GL34 mixture (1 mg mL^−1^) was incubated for 24 h with different carbohydrate degrading enzymes (5 U mL^−1^): α-amylase 1 (porcine pancreas); α-amylase 2 (*Aspergillus oryzae*); α-glucosidase (yeast); Iso-amylase (*Pseudomonas* sp.; pullulanase type 1 (*Klebsiella planticola*); β-galactosidase 1 (*A. oryzae*) and β-galactosidase 2 (*Kluyveromyces lactis*) (see Table S1 with detailed information).

### Intracellular and extracellular activity essays

After growth with GL34 as their only carbon source, the three tested *Bifidobacterium* strains were harvested by centrifugation at 10,000×*g* for 15 min at room temperature. Culture supernatants were sterilized using 0.45-μm filters and concentrated 10 times by Amicon Ultra-4 centrifugal filter units (10,000-Da molecular weight cutoff, Millipore). The harvested cell pellets were washed twice with 0.1 M potassium phosphate buffer (pH 6.6) and then suspended in 1 mL of this buffer into 2.0-mL screw-cap microtubes containing 400 mg of 0.1-mm-diameter glass beads (Biospec Products). Cell disruptions were carried out by homogenization by a mini bead-beater (Biospec Products) at 4200 rpm for six 1-min cycles with 40-s cooling on ice in between. The cytoplasmic extracts were harvested by centrifugation at 10,000×*g* for 5 min to remove cell wall fragments, and then concentrated to one fifth of the initial volume using Amicon Ultra-4 units as above.

The concentrated cell-free supernatants and cytoplasmic extracts (10 μg protein for each) were incubated separately with 5 mg mL^−1^ of the GL34 mixture. All reactions were performed in Milli-Q at 37 °C for 24 h. The progress of the reactions was followed by high-performance anion-exchange chromatography (HPAEC).

### High-pH anion-exchange chromatography

Samples were analyzed on an ICS-3000 workstation (Dionex, Amsterdam, the Netherlands) equipped with an ICS-3000 pulse amperometric detection (PAD) system and a CarboPac PA-1 column (250 × 2 mm; Dionex). The analytical separation was performed at a flow rate of 0.25 mL min^−1^ using a complex gradient of eluents A (100 mM NaOH); B (600 mM NaOAc in 100 mM NaOH); C (Milli-Q water); and D 50 mM NaOAc. The gradient started with 10% A, 85% C, and 5% D in 25 min to 40% A, 10% C, and 50% D, followed by a 35-min gradient to 75% A, 25% B, directly followed by 5-min washing with 100% B and reconditioning for 7 min with 10% A, 85% B, and 5% D. External standards of lactose, glucose, and fructose were used for calibration. For the determination of glucosylated lactose compounds with a degree of polymerization (DP) of 3, maltotriose was used as external standard.

### Bioinformatic analysis

All protein sequences from *B. adolescentis* ATCC 15703, *B. longum* subsp. *infantis* ATCC 15697, *B. breve* DSM 20123, and *B. breve* UCC 2003 used in this study were extracted from the National Center for Biotechnology Information (NCBI) database. Database searches used the non-redundant sequence database accessible at the NCBI website (http://www.ncbi.nlm.nih.gov) using BLASTP and global align search. The BLASTP searches and multiple-sequence alignments were used to find similarity between the characterized glucosidases of *B. breve* UCC 2003 and annotated glucosidases encoded by the studied bifidobacterial strains. Annotation of carbohydrate-active enzymes encoded by the genome sequences of *L. reuteri* 121 and *L. acidophilus* ATCC 4356 was carried out using dbCAN (http://csbl.bmb.uga.edu/dbCAN).

## Results

### Enzymatic hydrolysis of compounds in the GL34 mixture

**Fig. 1:**
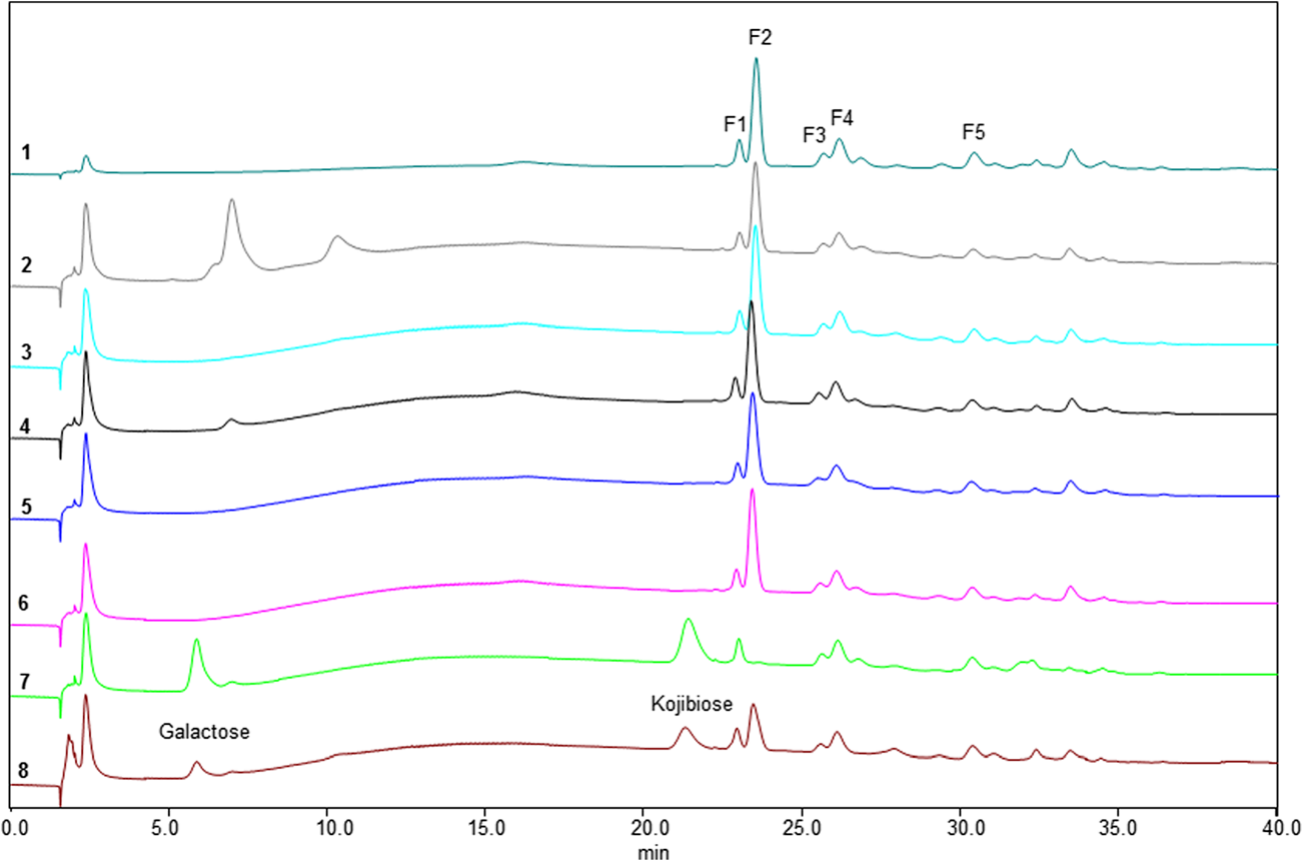
HPAEC profiles of oligosaccharides in 1 = the GL34 mixture (1 mg mL^−**1**^, blank) and the hydrolysis products after incubation of GL34 with 2 = α-amylase from porcine; 3 = α-amylase from *A. oryzae*; 4 = α-glucosidase from yeast; 5 = iso-amylase from *Pseudomonas* sp*.*; 6 = pullulanase type 1 from *K. planticola*; 7 = β-galactosidase from *A. oryzae* and 8 = β-galactosidase from *K. lactis*

The GL34 mixture of five compounds was synthesized using glucansucrase Gtf180-ΔN, decorating lactose with one or two glucose units from sucrose as donor substrate, also introducing different types of linkages (Pham et al. 2017). GL34 contains three DP3 compounds and two DP4 compounds, i.e., F1 (4′-glc-lac): α-D-Glc*p*-(1 → 4)-β-D-Gal*p*-(1 → 4)-D-Glc*p*; F2 (2-glc-lac): α-D-Glc*p*-(1 → 2)-[β-D-Gal*p*-(1 → 4)-]D-Glc*p*; F3 (3′-glc-lac): α-D-Glc*p*-(1 → 3)-β-D-Gal*p*-(1 → 4)-D-Glc*p*; F4 (4′,2-glc-lac): α-D-Glc*p*-(1 → 4)-β-D-Gal*p*-(1 → 4)-[α-D-Glc*p*-(1 → 2)-]D-Glc*p*; and F5 (3′,2-glc-lac): α-D-Glc*p*-(1 → 3)-β-D-Gal*p*-(1 → 4)-[α-D-Glc*p*-(1 → 2)-]D-Glc*p* (^4^). Four types of glycosidic linkages thus occur in this mixture, namely α(1 → 2), α(1 → 3), α(1 → 4), and β(1 → 4). Only the F2 2-glc-lac compound had been described before (Díez-Municio et al. 2012). The GL34 mixture also contains glucosyl residues linked α(1 → 3)/α(1 → 4) to the galactosyl residue of the original lactose. In view of the novel composition of this mixture of glucosylated-lactose compounds, we tested their resistance or sensitivity to hydrolysis with several commercially available enzymes. Following incubations with the porcine pancreas and *A. oryzae* α-amylases (Table S1), the HPAEC profiles at times 0 and 24 h showed no degradation of the GL34 compounds (Fig. 1). Also various malto-oligosaccharide acting enzymes (α-glucosidase, iso-amylase and pullulanase, Table S1) were tested for their ability to hydrolyze GL34 compounds. However, after 24-h incubation, no (monomeric or dimeric) products were detected in the reaction mixtures with these three enzymes (Fig. 1). None of these α-glucose cleaving enzymes thus was active on the GL34 compounds.

Subsequent incubation of the GL34 mixture with the β-galactosidase enzymes from *A. oryzae* and *K. lactis* (Table S1) however, did result in (some) hydrolysis. Fig. 1 shows that galactose and kojibiose (a glucose disaccharide with α(1 → 2)-linkage) were released during incubation with β-galactosidase, especially with the *A. oryzae* enzyme. Only the peak corresponding to F2 2-glc-lac disappeared, the only GL34 compound with a terminal galactosyl residue. We subsequently studied the utilization of these GL34 compounds for growth by (selected) common intestinal bacteria in more detail.

### Growth of human gut bacteria on the GL34 mixture

#### Fermentation of GL34 compounds by probiotic *Lactobacillus* strains

**Fig. 2:**
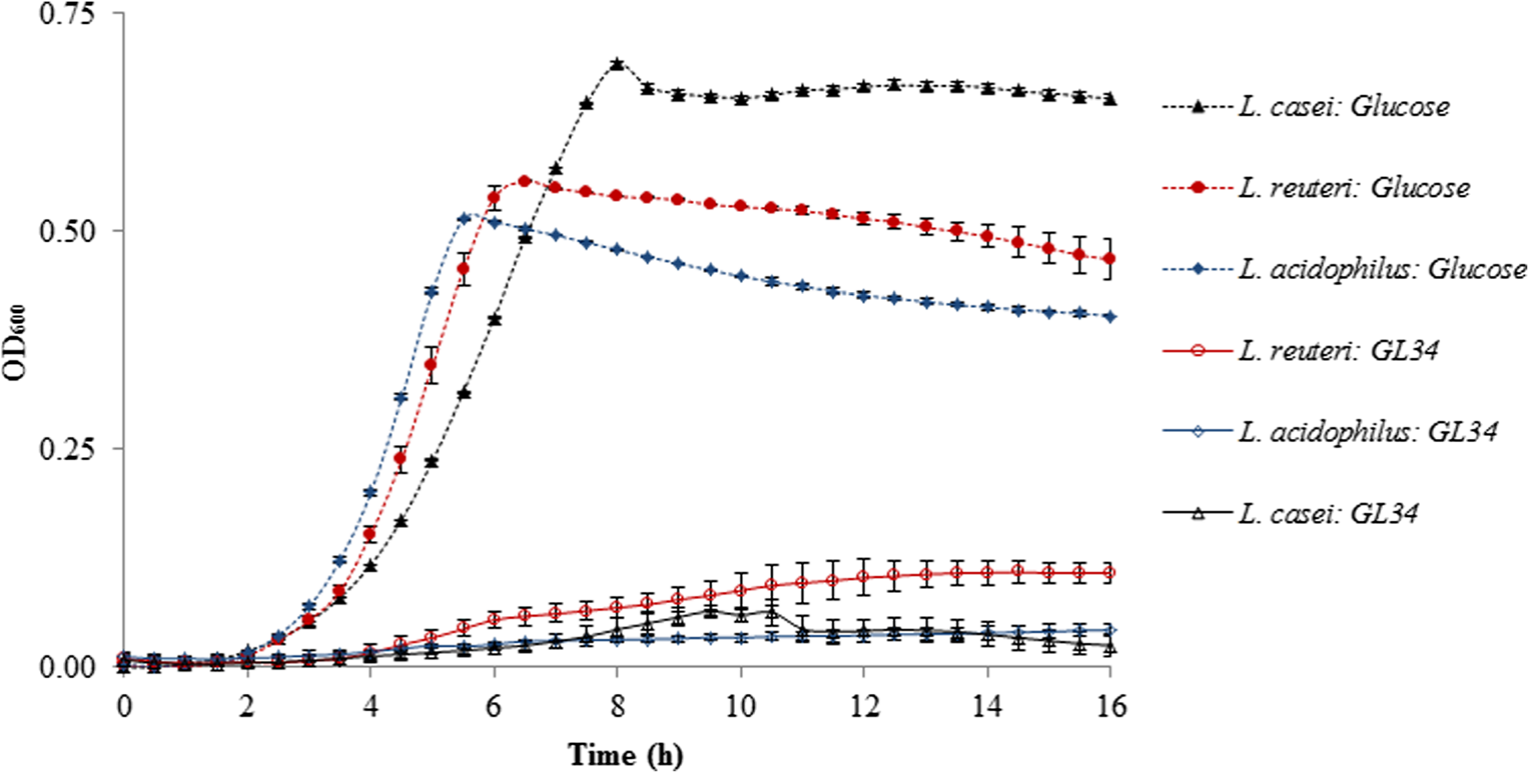
Growth of *L. casei* W56, *L. reuteri* 121, and *L. acidophilus* ATCC 4356 on GL34 compounds (5 mg mL^−**1**^). Glucose (5 mg mL^−**1**^) served as positive control; growth studies were carried out in triplicate

In this study, we tested *L. casei* W56, *L. acidophilus* ATCC 4356, and *L. reuteri* 121 and observed that all three strains showed limited growth on media with the GL34 mixture as the only carbon source (5 mg mL^−1^): the final (relative) OD_600_ values reached were 3.8%, 10.4%, and 26.5%, respectively, compared to a 100% control grown on glucose (Fig. 2).

#### Fermentation of GL34 compounds by probiotic *Bifidobacterium* strains

The GL34 mixture (5 mg mL^−1^) showed different stimulatory effects on the growth of *B. breve* DSM 20123, *B. adolescentis* ATCC 15703, and *B. longum* subsp. *infantis* ATCC 15697. *B. adolescentis* grew very well on GL34, its final OD_600_ value reached 80% of that of a 100% control growing on lactose, the purified TS0903 GOS mixture, and the GOS/FOS mixture (Table 1). However, the final OD_600_ values for growth of *B. breve* DSM 20123 and *B. longum* subsp. *infantis* ATCC 15697 on GL34 remained below 50% of the values for growth on lactose, the purified TS0903 GOS mixture and the GOS/FOS mixture (Table 1).

**Table 1:** Growth of *B. adolescentis* ATCC 15703, *B. longum* subsp. *infantis* ATCC 15697 and *B. breve* DSM 20123 on different carbon sources (5 mg mL^−**1**^) for 36 h, and the final pH values

Carbon sources	*Bifidobacterium adolescentis* ATCC 15703			*Bifidobacterium longum* subsp. *infantis* ATCC 15697			*Bifidobacterium breve* DSM 20213		
	OD_600_	%	pH	OD_600_	%	pH	OD_600_	%	pH
Lactose	2.00 ± 0.00	100	5.0	2.00 ± 0.00	100	5.0	2.00 ± 0.00	100	5.0
GOS	2.00 ± 0.00	100	5.0	1.77 ± 0.02	89	5.2	1.64 ± 0.04	82	5.3
GOS/FOS	2.00 ± 0.00	100	4.9	1.79 ± 0.02	90	5.3	1.61 ± 0.01	81	5.2
GL34	1.60 ± 0.03	80	5.0	0.73 ± 0.03	37	5.3	0.86 ± 0.04	43	5.4

The experiments were carried out in triplicate, and the average values are shown

**Fig. 3:**
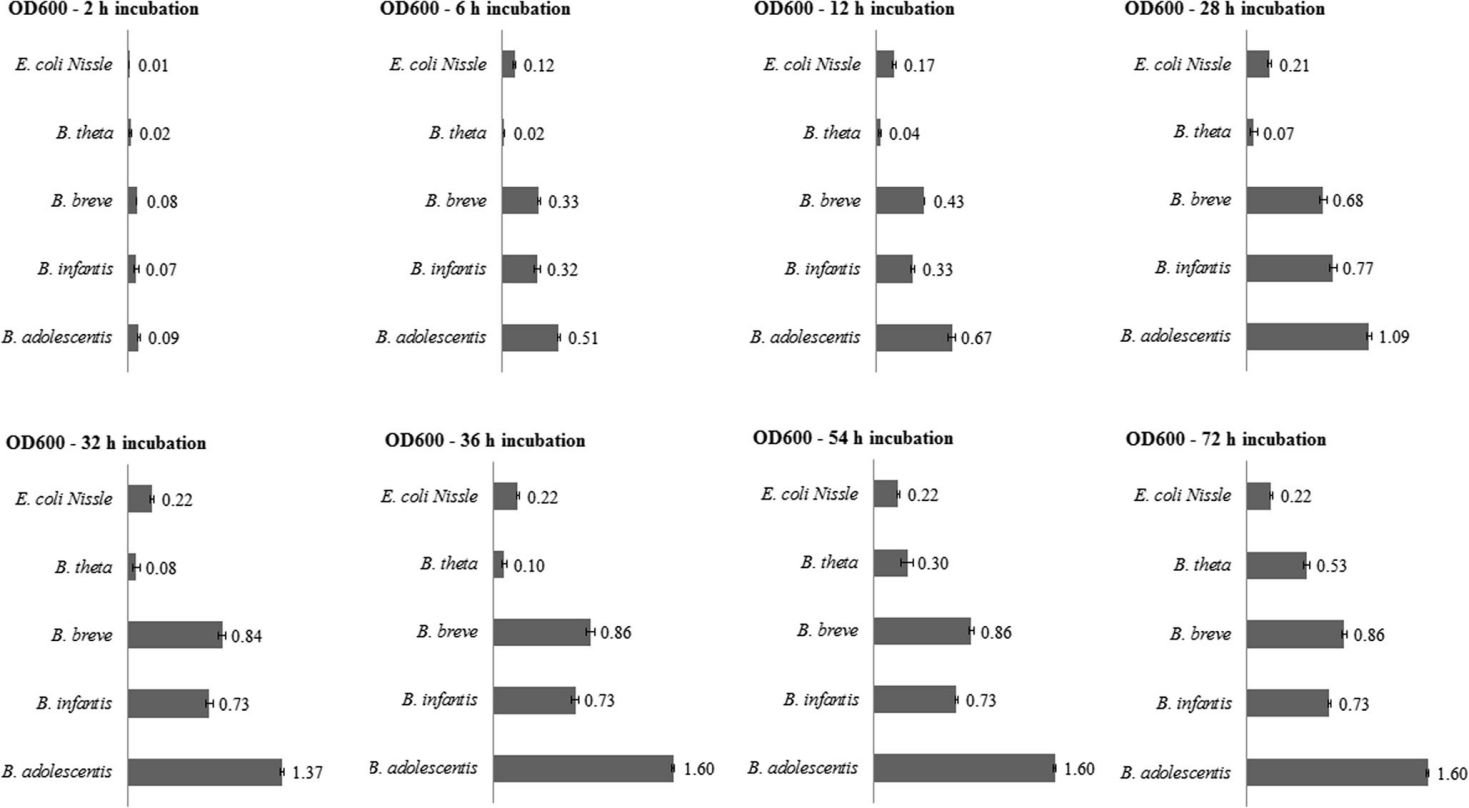
Growth of *B. adolescentis* ATCC 15703, *B. longum* subsp. *infantis* ATCC 15697, *B. breve* DSM 20123, *E. coli* Nissle, *B. thetaiotaomicron* in medium supplemented with 5 mg mL^−1^ GL34 at different times of incubation (h). Growth experiments were carried out in triplicate

The tested bifidobacterial strains displayed two or more growth phases (Fig. 3 and Fig. S1). Some compounds in the GL34 mixture thus are more preferred growth substrates than others. *B. breve* and *B. longum* subsp. *infantis* grew more slowly on GL34 than *B. adolescentis*. They reached OD values around 0.70 after 24 h incubation compared to 12 h for *B. adolescentis*. Final maximal OD values were 0.86 for *B. breve* and 0.73 for *B. longum* subsp. *infantis* and 1.60 for *B. adolescentis.* The latter strain appeared to go through different lag phases, adapting to the different carbon sources in GL34, reaching maximal OD after 36 h of incubation (Fig. S1).

#### Fermentation of GL34 compounds by commensal gut bacteria

**Fig. 4:**
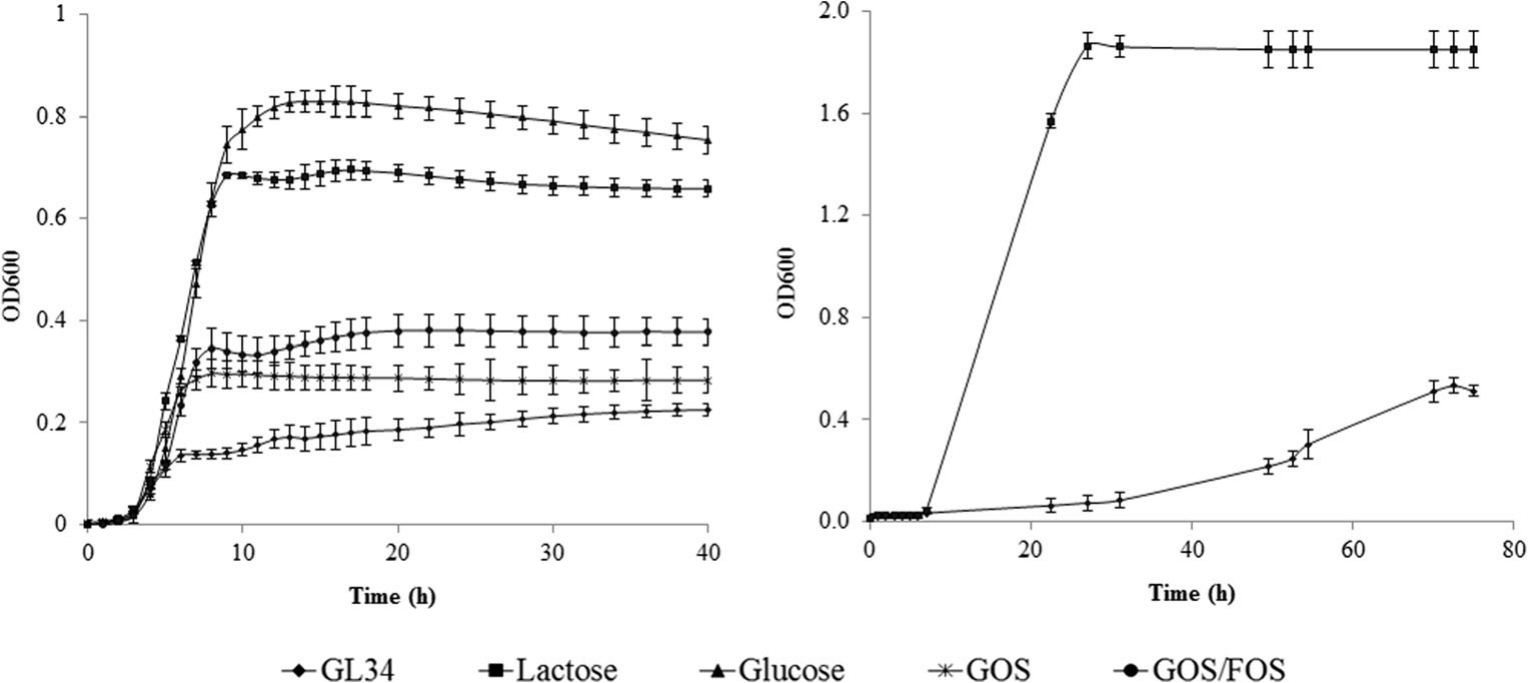
**1** Growth of *E. coli* Nissle on the GL34, TS0903 GOS, and GOS/FOS mixtures (5 mg mL^−**1**^); lactose and glucose (5 mg mL^−**1**^) served as positive controls. **2** Growth of *B. thetaiotaomicron* on the GL34 compounds (5 mg mL^−**1**^); lactose (5 mg mL^−**1**^) served as positive control. Growth studies were carried out in triplicate

Also, the ability of two selected commensal bacteria to grow on the GL34 mixture was studied. *B. thetaiotaomicron* is a Gram-negative anaerobic bacterium found dominantly in human distal intestinal microbiota (Xu et al. 2003). *E. coli* Nissle represents ecologically important inhabitants of the human intestinal tract (Mason and Richardson 1981). Growth of *E. coli* Nissle on the GL34 mixture, TS0903 GOS and a GOS/FOS mixture (5 mg mL^−1^), was relatively minor with final OD_600_ values of 0.19 ÷ 0.35 after 24-h of incubation, compared to growth on lactose and glucose with final OD_600_ values of 0.66 and 0.74, respectively (Fig. 4, panel 1). The final OD_600_ value of *B. thetaiotaomicron* was 0.53 after 72 h of incubation, but its growth displayed a pronounced lag phase (Fig. 4, panel 2). This strain thus may fail to compete with other bacteria which have shorter lag phases of growth with the GL34 mixture, such as the bifidobacteria tested and *E. coli* Nissle.

We subsequently identified the specific GL34 compounds utilized by these strains, and products derived, also aiming to elucidate which hydrolytic enzyme activities are involved, with emphasis on β-galactosidases and α-glucosidases.

### Hydrolytic activity of commensal bacteria and lactobacilli on the GL34 mixture

**Fig. 5:**
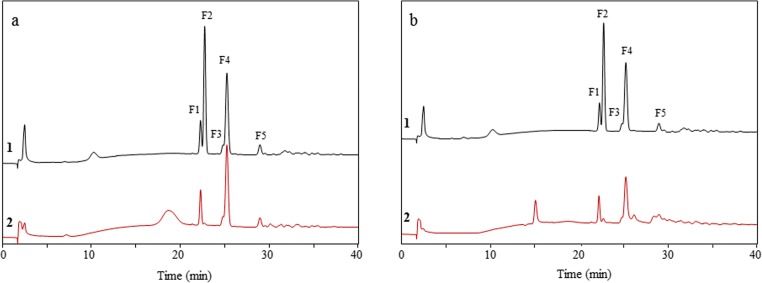
HPAEC profiles of oligosaccharides in 1 = the GL34 mixture (1 mg mL^−1^, blank) and 2 = the hydrolysis products of GL34 fermentation by **a***E. coli* Nissle at 40 hand **b***B. thetaiotaomicron* at 72 h

The GL34 compounds can be visualized as individual peaks in HPAEC-PAD chromatograms. The compounds consumed by the tested bacteria were validated by peak disappearance at the corresponding retention time. HPAEC analysis of culture supernatants of the commensal bacteria grown on the GL34 mixture showed that the F2 peak corresponding to 2-glc-lac disappeared completely (Fig. 5), indicating the selective and full utilization of only F2. Most likely this is based on β-galactosidase degradation of F2 2-glc-lac, followed by α-glucosidase degradation of the kojibiose formed, and finally, consumption of the galactose and glucose formed for cellular growth. Only in the case of *E. coli* Nissle degradation resulted in accumulation of kojibiose. This was not investigated any further. Besides, *B. thetaiotaomicron* was also able to degrade F4 4′,2-glc-lac partially (Fig. 5).

**Fig. 6:**
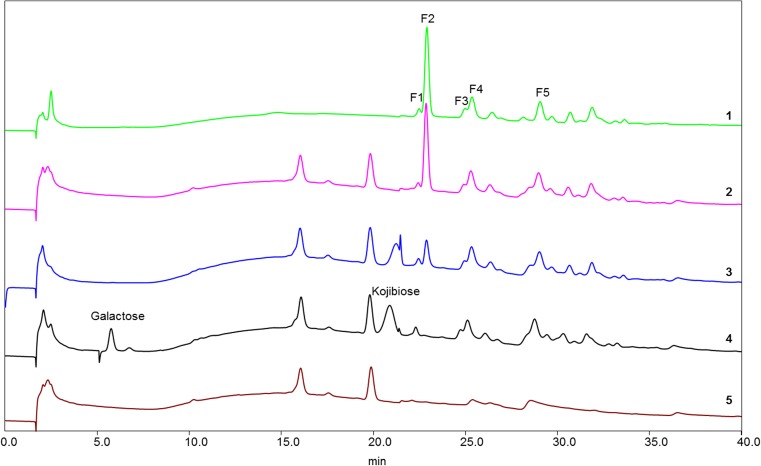
HPAEC profiles of oligosaccharides in 1 = the GL34 mixture (1 mg mL^−1^, blank) and the hydrolysis products of GL34 fermentation after 16 h of incubation with 2 = *L. casei* W56; 3 = *L. acidophilus* ATCC 4356; 4 = *L. reuteri* 121, and 5 = MRS medium (blank)

HPAEC profiles of *L. casei* W56 culture supernatants showed that none of the GL34 compounds were degraded (Fig. 6), in line with the very limited growth observed (Fig. 2). When searching the annotated genome sequence of this strain in the CAZy database (http://www.cazy.org/b7858.html), we did not find any (putative) β-galactosidase. This most likely explains the inability of *L. casei* W56 to hydrolyze the galactose β(1 → 4) linked to glucosyl residue in F2 2-glc-lac. Many putative α-glucosidases were found encoded in the genome sequence of this strain, however. In view of the results obtained, these enzymes apparently are inactive on the GL34 compounds, or the GL34 mixture is unable to induce their expression. This was not investigated further.

*Lactobacillus acidophilus* ATCC 4356 and *L. reuteri* 121 strains only showed hydrolytic activity with F2 2-glc-lac in the GL34 mixture. *L. reuteri* 121 degraded this compound to a higher level than *L. acidophilus* ATCC 4356 (Fig. 6). Annotation of carbohydrate-active enzymes encoded by the genome sequence of *L. reuteri* 121 (Gangoiti et al. 2017) was carried out using dbCAN (http://csbl.bmb.uga.edu/dbCAN). One putative β-galactosidase of family GH2 was detected (gene number: BJI45_06415), which may be responsible for hydrolysis of F2. β-Galactosidases of this GH2 family are known to hydrolyze a wide variety of β-(1 → 2, 3, 4, or 6) GOS, including oligosaccharides with a degree of polymerization of 3–6 (Gänzle 2012). Kojibiose released from the F2 compound by this strain remained in the medium without being degraded (completely). Only a single (predicted) extracellular α-glucosidase of family GH31 was encoded in the genome of *L. reuteri* 121 (gene number: BIJ45_02455) (Gangoiti et al. 2017). This enzyme is apparently unable to effectively degrade kojibiose. Apparently, there is also no transporter expressed that can facilitate the uptake of kojibiose for intracellular degradation.

*Lactobacillus acidophilus* ATCC 4356 is known to encode a β-galactosidase (LacZ, classified in the GH2 family) (Gänzle 2012; Schwab et al. 2010; Pan et al. 2010).This LacZ enzyme thus may be responsible for degradation of the β(1 → 4)-linkage in F2 and release of kojibiose. Annotation of carbohydrate-active enzymes encoded by the genome sequence of *L. acidophilus* ATCC 4356 (Palomino et al. 2015) was also carried out by dbCAN (http://csbl.bmb.uga.edu/dbCAN). Ten putative α-glucosidase enzymes (family GH13 and GH31) were annotated in the *L. acidophilus* ATCC 4356 genome. Agl3 is anα-glucosidase identified in *B. breve* UCC 2003, with a broad hydrolytic activity towards all possible α-glycosidic linkages, including the α(1 → 2) linkage in sucrose and kojibiose (Kelly et al. 2016). However, a BLAST analysis of these putative glucosidases of *L. acidophilus* ATCC 4356 and Alg3 showed very low similarity in protein sequence (between 24 and 31%) (Table S2). The observed accumulation of kojibiose in the growth medium of *L. acidophilus* ATCC 4356 (Fig. 6) thus may be due to lack of an extracellular α-glucosidase active on α(1 → 2) linkages. Furthermore, it is possible that the α-glucosidase enzymes of this strain are only intracellular enzymes and that a suitable transporter for kojibiose is absent.

### Hydrolytic activity of the *Bifidobacterium* strains on the GL34 mixture

Typically, bifidobacteria have extracellular endohydrolases acting on glycosidic bonds of oligo- and polymeric substrates, yielding smaller products which are internalized by carbohydrate-specific (ABC type) transporters. Further utilization may be carried out by cytoplasmic GHs such as α/β-glucosidases and α/β-galactosidases to produce monosaccharides which are used for growth (O’Connell-Motherway et al. 2011; O’Connell et al. 2013). To try and identify the bifidobacterial enzymes responsible for utilization of GL34 compounds, cells grown in modified BM broth containing 5 mg mL^−1^ GL34 were harvested at 36 h (Fig. 3). Three fractions were prepared, namely growth culture supernatants, concentrated culture supernatants and cytoplasmic extracts (see “Materials and methods”). GL34 was incubated with concentrated culture supernatants and cytoplasmic extracts to verify the presence or absence of extra- and/or intracellular enzyme activities involved in degradation.

### *Bifidobacterium adolescentis* ATCC 15703

**Fig. 7:**
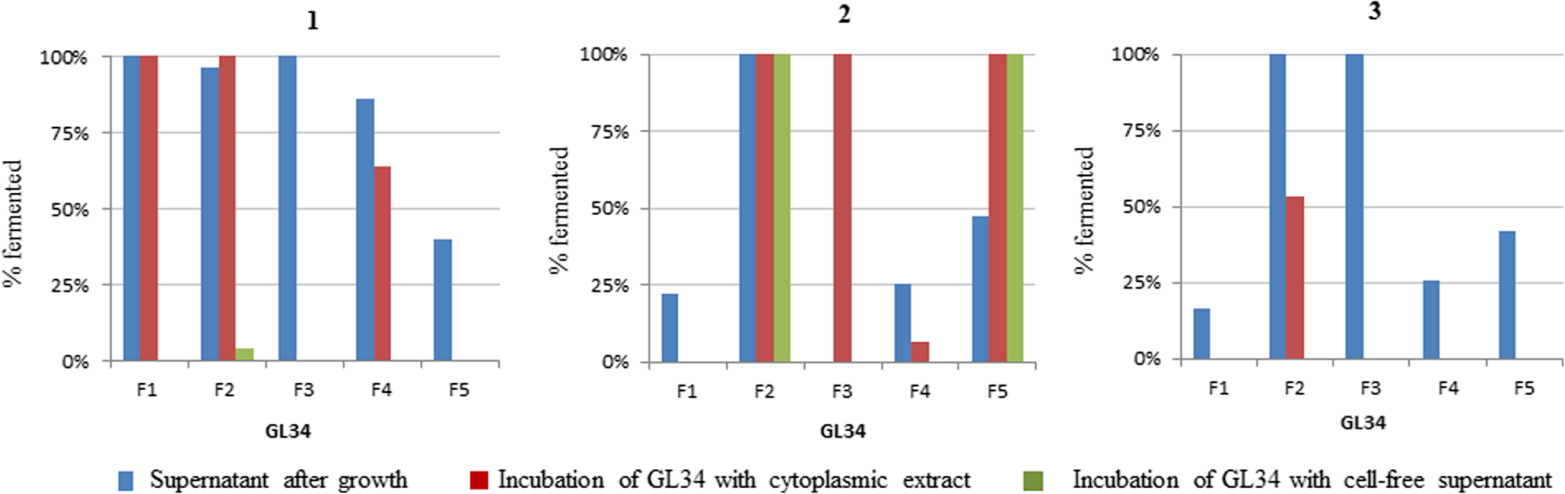
Consumption of GL34 compounds (%) during growth of **1***B. adolescentis* ATCC 15703; **2***B. longum* subsp. *infantis* ATCC 15697; and **3***B. breve* DSM 20213, and after 24-h incubations of 5 mg mL^−**1**^ GL34 with cytoplasmic extracts or cell-free supernatants (obtained after growth of these three strains on the GL34 mixture)

*Bifidobacterium adolescentis* was able to utilize all GL34 compounds, and only 10 and 60% of F4 4′,2-glc-lac and F5 3′,2-glc-lac remained, respectively (Fig. 7, panel 1). This also explains the relatively strong growth of this strain (Fig. 3). None of the GL34 compounds were hydrolyzed by cell-free culture supernatants of this strain. Its cell extracts only hydrolyzed F1 4′-glc-lac, F2 2-glc-lac, and F4 4′,2-glc-lac compounds (Fig. 7, panel 1). Three α-glucosidases are annotated in the *B. adolescentis* ATCC 15703 genome sequence (http://www.cazy.org), namely AglB; AglA and BAD-0971. AglB exhibited a preference for hydrolyzing α(1 → 4) linkages in maltose (van den Broek et al. 2003), and most likely also is involved in cleavage of the α(1 → 4) linkages present in the F1 and F4 compounds. AglA showed high hydrolytic activity only towards α(1 → 6) linkages in isomaltotriose and minor activity towards α(1 → 1) linkages in trehalose (van den Broek et al. 2003). None of these two enzymes are able to degrade α(1 → 3) linkages occurring in F3 3′-glc-lac and F5 3′,2-glc-lac. It thus remained possible that during growth the F3 and F5 were (partially) degraded by the BAD-0971 enzyme, the third *B. adolescentis* ATCC 15703 α-glucosidase.

The BAD-0971 protein has been annotated as an α-1,4-glucosidase but has not been characterized yet (Suzuki et al. 2006). A BLASTP analysis was carried out to compare its protein sequence with those of the characterized glucosidases of *B. adolescentis* ATCC 15703 (AglA and AglB) and *B. breve* UCC 2003 (Agl1; Agl2 and Agl3). Agl3 from *B. breve* UCC 2003, as mentioned above, has a broad hydrolytic specificity towards α(1 → 2), α(1 → 3), α(1 → 4), α(1 → 5), and α(1 → 6) in kojibiose, turanose, maltose, leucrose, and palatinose, respectively (McLaughlin et al. 2015). The two other α-glucosidases of this strain (Agl1 and Agl2) are more active in cleaving α(1 → 6) linkages (in panose, isomaltose, isomaltotriose, and palatinose) and less efficient in cleaving α(1 → 3) linkages in turanose and nigerose, α(1 → 4) in maltulose and α(1↔2) in sucrose (Pokusaeva et al. 2009). AglA and Agl3 exhibit 59% and 73% similarity/identity, respectively, to the BAD-0971 protein of *B. adolescentis* (Table S3). The BAD-0971 enzyme may have a broad hydrolytic specificity similar to Agl3. However F3 3′-glc-lac and F5 3′,2-glc-lac with α(1 → 3) linkages remained undigested in cell extracts. It cannot be excluded that the cell disruption process has weakened activity of BAD-0971 (annotated as an α-1,4-glucosidase), or other hitherto unidentified α-glucosidase enzymes.

### *Bifidobacterium longum* subsp. *infantis* ATCC 15697

The F2 2-glc-lac and F3 3′-glc-lac compounds were completely utilized during growth of *B. longum* subsp. *infantis*, and the F1 4′-glc-lac, F4 4′,2-glc-lac and F5 3′,2-glc-lac compounds only for 20–40% (Fig. 7, panel 2). Cell extracts only hydrolyzed F2 2-glc-lac, apparently involving (an) intracellular enzyme(s) (Fig. 7, panel 2). The F1 4′-glc-lac, F3 3′-glc-lac, F4 4′,2-glc-lacand F5 3′,2-glc-lac structures were not utilized by cell extracts of this strain, suggesting that it lacks α-glucosidase enzymes acting on glucose α(1 → 3)/α(1 → 4) linked to galactosyl residue. The complete use of the F3 3′-glc-lac compound during growth of *B. longum* subsp. *infantis* ATCC 15697 thus remained unexplained again. No extracellular activity of *B. longum* subsp. *infantis* towards the GL34 mixture was detected (Fig. 7, panel 2). Its limited activity on the GL34 mixture resulted in the lowest growth extent (37%) compared to the other two bifidobacteria (Table 1). Various glucosidases have been annotated in the genome of *B. longum* subsp. *infantis* mainly with activity on α(1 → 4) and α(1 → 6) glucosidic linkages, namely α-1,4 glucosidase (locus tag BLIJ-0129); putative amylase (locus tag BLIJ-0286); putative iso-amylase (locus tag BLIJ-0286 and BLIJ-2315); and oligo-1,6-glucosidase (locus tag BLIJ-2526) (www.cazy.org/). However, BLAST analysis showed very low similarity in their protein sequence between the annotated glucosidases from this strain and Alg3 (Table S4). This most likely explains the poor growth of this strain on the GL34 mixture, and its limited intracellular activity on α1 → 3/α1 → 4 glucosidic linkages in F1 4′-glc-lac, F3 3′-glc-lac, F4 4′,2-glc-lac, and F5 3′,2-glc-lac.

### *Bifidobacterium breve* DSM 20213

In case of *B. breve*, four of the five GL34 compounds (F1 4′-glc-lac, F3 3′-glc-lac, F4 4′,2-glc-lac, and F5 3′,2-glc-lac) (partly) remained unutilized in culture supernatants after growth (Fig. 7, panel 3). Amongst the three bifidobacteria tested, only *B. breve* possessed extracellular activity on the mixture GL34: the F2 2-glc-lac and F5 3′,2-glc-lac compounds were completely degraded by cell-free supernatants after 24-h incubation (Fig. 7, panel 3). The extracellular activity was also observed previously in different *B. breve* strains; however, on α(1 → 4) and α(1 → 6) glucosidic linkages present in starch, amylopectin and pullulan (Ryan et al. 2006). Nevertheless, cell extracts fully hydrolyzed F2 2-glc-lac, F3 3′-glc-lac, and F5 3′,2-glc-lac (Fig. 7, panel 3). Most likely *B. breve* is unable to transport F3 3′-glc-lac and F5 3′,2-glc-lac into the cell. These data suggest that intracellular enzymes of this strain were able to cleave off the glucose units α(1 → 3) linked to the galactosyl residues occurring in F3 3′-glc-lac and F5 3′,2-glc-lac but not the glucose units α(1 → 4) linked to the galactosyl residues in F1 4′-glc-lac and F4 4′,2-glc-lac.

The putative α-glucosidases in the *B. breve* DSM 20213 genome, however, are annotated to act mainly on α(1 → 4) and α(1 → 6) glucosidic linkages; namely α-1,4 glucosidase (locus tag BBBR-0095); putative amylase (locus tags BBBR-0101; BBBR-0825 and BBBR-0257) and oligo-1,6-glucosidase (locus tags BBBR-0484 and BBBR-1863) (www.cazy.org/). This uncharacterized α-1,4 glucosidase (locus tag BBBR-0095) was found to have 99% similarity with Alg3 of *B. breve* UCC 2003 (Table S4). This annotated α-1,4 glucosidase thus also may be able to hydrolyze α(1 → 3) linkage in F3 3′-glc-lac and F5 3′,2-glc-lac. Structures of F1 4′-glc-lac and F4 4′,2-glc-lac may be inaccessible to this α-glucosidase, or it is unable to cleave their α(1 → 4) linkages, thus explaining their (very) limited degradation by *B. breve* DSM 20213.

The annotated β-galactosidases (family GH2) of these three *Bifidobacterium* strains are active on the F2 2-glc-lac compound, containing a galactosyl-moiety with a β(1 → 4) linkage to kojibiose. Following growth on GL34 as only carbon source, all three strains exhibited clear intracellular activity with F2 2-glc-lac. The data showed that individual *Bifidobacterium* strains have preference for degradation of glucosylated lactose compounds with α(1 → 3) and α(1 → 4) glucosidic linkages.

## Discussion

The GL34 compounds generally consist of a lactose molecule at the reducing end that is elongated with one or more glucose molecules involving different linkage types (Pham et al. 2017). This combination of different monomers, i.e., glucose and galactose, with various linkage types (α(1 → 2), α(1 → 3), α(1 → 4), and β(1 → 4)) and different degrees of polymerization, increases the diversity of lactose-derived oligosaccharides available. The GL34 compounds were resistant to degradation by the α-amylases of porcine pancreas and *A. oryzae*. These are endo-acting enzymes (EC 3.2.1.1), degrading α(1 → 4) glucosidic linkages in polysaccharides such as starch or glycogen (van der Maarel et al. 2002), mainly yielding glucose and maltose. Also, various MOS-acting enzymes were tested: The α-glucosidase enzyme of yeast used is known as an exo-acting enzyme, hydrolyzing α(1 → 4) glucosidic linkages but only at the terminal non-reducing (1 → 4)-linked α-glucose residues of di- and oligosaccharides to release a single glucose unit (Reese et al. 1968). Iso-amylase from *Pseudomonas* sp. is an α(1 → 4,6) debranching enzyme (van der Maarel et al. 2002). Pullulanase type I of *K. planticola* hydrolyzes α(1 → 6) glucosidic linkages in pullulan and starch (Domań-Pytka and Bardowski 2004). Also, these three enzymes failed to cleave any compounds in the GL34 mixture. Incubations with β-galactosidase enzymes however did result in hydrolysis, but only the F2 2-glc-lac molecule disappeared. This is explained by the ability of these enzymes to catalyze hydrolysis of β-glycosidic bonds between galactose and its organic moiety. The combined data thus shows that the GL34 compounds are (largely) resistant to hydrolysis by these common carbohydrate degrading enzymes (Fig. 1).

There is abundant clinical evidence for the important roles of *Bifidobacterium* and *Lactobacillus* species in the eco-physiology of the intestinal microbiota (Picard et al. 2005; Sanders and Klaenhammer 2001), and different strains of lactobacilli are marketed as commercial probiotics. Individual bifidobacteria are known to have specific substrate preferences (Degnan and Macfarlane 1991). The GL34 mixture stimulated growth of bifidobacteria, but indeed to different extents. Only utilization of the F2 2-glc-lac compound has been studied previously and was shown to have a limited stimulatory effect on the growth of *B. breve* 26M2. This F2 compound did not stimulate growth of lactobacilli tested in our present study, as previously shown for *L. casei* LC-01 (García-Cayuela et al. 2014). However, we observed that F2 2-glc-lac stimulated growth of the probiotic bacteria *L. reuteri* 121, *B. adolescentis* ATCC 15703, *B. longum* subsp. *infantis* ATCC 15697, and *B. breve* DSM 20213, and also of two commensal bacteria, *E. coli* Nissle and *B. thetaiotaomicron*, albeit to various extents. This F2 compound thus is less selective in comparison with the other compounds in the GL34 mixture*.* Both F1 4′-glc-lac and F4 4′,2-glc-lac also stimulated growth of all three tested bifidobacteria, again to various extents. The presence of an α(1 → 3) linkage makes F3 3′-glc-lac more selective than F1 and F4: F3 was utilized by only two out of three studied *Bifidobacterium* strains: *B. adolescentis* ATCC 15703 and *B. breve* DSM 20213. Also, F5 3′,2-glc-lac with both α(1 → 2) and α(1 → 3) glucosidic linkages showed similar stimulatory effects on all three *Bifidobacterium* strains.

In conclusion, the GL34 mixture promotes growth of the tested bacteria to different extents. The bifidobacteria tested generally were better at degrading GL34 compounds than the lactobacilli and commensal bacteria. The stronger metabolic toolset of bifidobacteria in comparison with lactobacilli also has been observed when comparing their growth on human milk oligosaccharides and other prebiotic oligosaccharides as primary carbon source (Watson et al. 2013; Sela et al. 2008; González et al. 2008; McLaughlin et al. 2015). The GL34 mixture thus showed potential to shift microbiota composition by specifically stimulating growth of bifidobacteria, particularly *B. adolescentis*. Four out of five compounds in this GL34 mixture exerted high and selective growth stimulatory effects towards health-beneficial probiotic bifidobacteria. The combination of monomer composition and linkage type clearly determines the fermentable properties of the GL34 compounds. Individual gut bacteria were able to utilize only specific compounds in the GL34 mixture. Synergistic activities between bacterial species thus are likely to be essential for the utilization of the whole GL34 mixture. In future work, this will be studied in more detail, e.g., by using fecal bacterial cultures. Only *B. adolescentis* was able to utilize almost all structures, providing a potential synbiotic combination.

## Electronic supplementary material

ESM 1 (PDF 120 kb)
