# Supplementary material for: Stimulatory effects of novel glucosylated lactose derivatives GL34 on growth of selected gut bacteria
Source: Appl Microbiol Biotechnol. 2018 Nov 7;103(2):707–18. doi: 10.1007/s00253-018-9473-8 (PMC6373440; doi:10.1007/s00253-018-9473-8)
Supplement: Supplementary file 1 — (PDF 120 kb) [file 253_2018_9473_MOESM1_ESM.pdf]

Supplementary material

Journal: Applied Microbiology and Biotechnology

Title: Stimulatory effects of novel glucosylated lactose derivatives GL34 on growth of selected gut bacteria

Authors: Hien T. T. Pham[1], Markus C.L. Boger[1], Lubbert Dijkhuizen[1,2,#] and Sander S. van Leeuwen[1,3]

Affiliated address:

[1]Microbial Physiology, Groningen Biomolecular Sciences and Biotechnology Institute (GBB), University of Groningen, Nijenborgh 7, 9747 AG Groningen, The Netherlands

[2]Current address: CarbExplore Research B.V., Zernikepark 12, 9747 AN Groningen, The Netherlands

[3]Current address: Department of Laboratory Medicine, University Medical Center Groningen, University of Groningen, 9713 GZ Groningen, The Netherlands

***Corresponding author:** Professor Lubbert Dijkhuizen, Ph.D.

Microbial Physiology, Groningen Biomolecular Sciences and Biotechnology Institute (GBB), University of Groningen, Nijenborgh 7, 9747 AG Groningen, The Netherlands

Phone: +31 50 36 32153; fax: +31 50 36 32154; E-mail: L.Dijkhuizen@rug.nl

Table S1: Carbohydrate acting enzymes used to analyze degradation of the GL34 compounds. The pH and temperature values used are shown.

Enzymes	Origins	Source	GH family	Reaction conditions	
				pH	Temperature °C
α-amylase 1	Porcine pancreas	Sigma-Aldrich	GH13	7.0	50
α-amylase 2	*Aspergillus oryzae*	Megazyme	GH13	5.0	50
α-glucosidase	Yeast	Megazyme	GH13	7.0	40
Iso-amylase	*Pseudomonas sp.*	Megazyme	GH13	4.0	40
Pullulanase type 1	*Klebsiella planticola*	Megazyme	GH13	5.0	40
β-galactosidase 1	*Aspergillus oryzae*	Megazyme	GH2	4.7	45
β-galactosidase 2	*Kluyveromyces lactis*	Megazyme	GH2	7.0	40

Table S2: Sequence similarity levels between Agl3 from *Bifidobacterium breve* UCC 2003 and putative α-glucosidases encoded in the *Lactobacillus acidophilus* ATCC 4356 genome.

NCBI accession number	Annotated GH family*	Query cover (%)	Identity level (%)
WP_003546237.1	GH31	35	23
WP_003548000.1	GH31	28	24
WP_003548741.1	GH31	21	23
WP_003549626.1	GH13	40	26
WP_003549917.1	GH13 GH31	28	30
WP_011254098.1	GH13 GH31	93	29
WP_011254225.1	GH13	46	26
WP_011254315.1	GH13	78	31
WP_011254601.1	GH13 GH31	96	28
WP_021721607.1	GH31	25	24

* Annotation of proteins from the genome sequence of *L. acidophilus* ATCC 4356 was carried out using dbCAN (http://csbl.bmb.uga.edu/dbCAN).

Table S3: Protein sequence similarity levels of BAD-0971 from *B. adolescentis* ATCC 15703 to annotated α-glucosidases in other bifidobacteria.

Protein name	Annotated or proven functions	Bacterial strain	NCBI accession number	Identity level (%)
AglA	α-1,6-glucosidase	*B. adolescentis* ATCC 15703	Query_123011	73
AglB	α-glucosidase	*B. adolescentis* ATCC 15703	Query_123011	73
Agl1	α-1,6-glucosidase	*B. breve* UCC 2003	Query_83439	30
Agl2	α-1,6-glucosidase	*B. breve* UCC 2003	Query_98509	32
Agl3	α-glucosidase	*B. breve* UCC 2003	Query_165455	59

Table S4: Similarity levels of GH13 glucosidase proteins from *B. longum* subsp. *infantis* ATCC 15697 and *B. breve* DSM 20123 to the annotated Alg3 from *B. breve* UCC 2003.

Bacterial strain	Locus tags	Annotated or proven functions	NCBI accession number	Identity level (%)
B. infantis ATCC 15697	BLIJ-0129	α-1,4-glucosidase	Query_130701	19
B. infantis ATCC 15697	BLIJ-0286	putative amylase	Query_56433	16
B. infantis ATCC 15697	BLIJ-1799	putative isoamylase	Query_179069	17
B. infantis ATCC 15697	BLIJ-2315	putative isoamylase	Query_117079	16
B. infantis ATCC 15697	BLIJ-2526	oligo-1,6-glucosidase	Query_140967	29
<u>*B. breve* DSM 20123</u>	<u>BBBR-0095</u>	<u>α-1,4-glucosidase</u>	<u>Query_5141</u>	<u>99</u>
B. breve DSM 20123	BBBR-0101	putative amylase	Query_163329	25
B. breve DSM 20123	BBBR-0257	putative amylase	Query_188975	17
B. breve DSM 20123	BBBR-0484	Oligo-1,6-glucosidase	Query_211369	29
B. breve DSM 20123	BBBR-0825	putative amylase	Query_229645	19
B. breve DSM 20123	BBBR-1863	Oligo-1,6-glucosidase	Query_8587	28

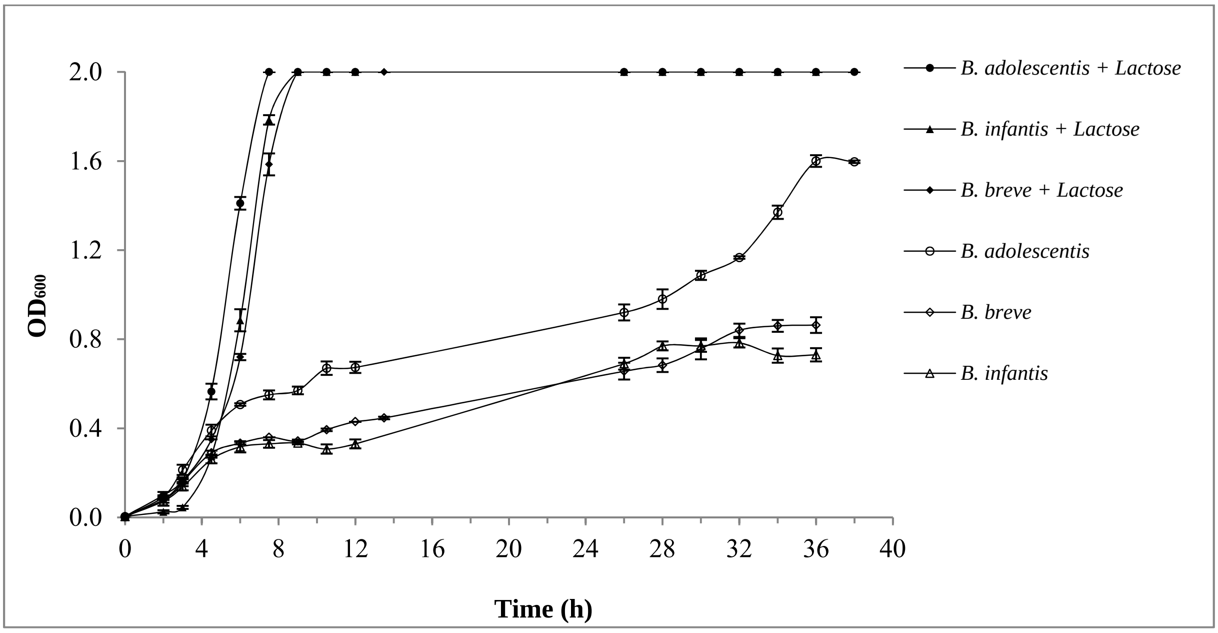

Fig. S1 Growth of *B. adolescentis* ATCC 15703, *B. infantis* ATCC 15697 and *B. breve* DSM 20123 on GL34 compounds (5 mg mL^{-1}). Lactose (5 mg mL^{-1}) served as positive control, growth studies were carried out in triplicate.
